# Meta-analyses of *IL1A* polymorphisms and the risk of several autoimmune diseases published in databases

**DOI:** 10.1371/journal.pone.0198693

**Published:** 2018-06-07

**Authors:** Hang Su, Na Rei, Lei Zhang, Jiaxiang Cheng

**Affiliations:** 1 The Second Department of Orthopedics, Cangzhou Central Hospital, Cangzhou, China; 2 The First Department of Gynecology, Cangzhou People’s Hospital, Cangzhou, China; South Texas Veterans Health Care System, UNITED STATES

## Abstract

**Background:**

Based on published data, we aimed to quantitatively elucidate the possible genetic influence of rs17561 G/T and rs1800587 C/T polymorphisms of the *IL1A* (interleukin 1 alpha) gene in the susceptibility to several autoimmune diseases.

**Methods:**

A series of meta-analyses were carried out. After database searching, we utilized our inclusion/exclusion criteria to screen and include the eligible studies. *P*_*association*_ (*P* value of association test), Bonferroni-corrected *P*_association_ value; false discovery rate (FDR)-corrected *P*_*association*_, ORs (odd ratios), and 95% CI (confidence interval) were generated to assess the magnitudes of genetic relationships.

**Results:**

A total of 35 eligible articles were included. Pooled analysis data of both rs17561 G/T and rs1800587 C/T in the overall population indicated a negative association between cases of autoimmune diseases and negative controls (all *P*_*association*_>0.05, Bonferroni-corrected *P*_association_>0.05, FDR-corrected *P*_*association*_>0.05). Similar results were found in most subgroup analyses (all *P*_*association*_>0.05, Bonferroni-corrected *P*_association_>0.05, FDR-corrected *P*_*association*_>0.05), apart from the rs1800587 in the Graves’ disease subgroup, which showed an increased risk in some cases, compared with controls, under the models of allele T vs. C, carrier T vs. C, CT+TT vs. CC, and CT vs. CC (all *P*_*association*_<0.05, Bonferroni-corrected *P*_association_<0.05, FDR-corrected *P*_*association*_>0.05, OR>1).

**Conclusion:**

Based on the available data, C/T genotype of the rs1800587 polymorphism within *IL1A gene* may be associated with an increased Graves’ disease risk. We did not see evidence regarding a positive role for rs1800587 or rs17561 in the risk of other autoimmune diseases, such as systemic sclerosis or rheumatoid arthritis. These conclusions still merit further data support and molecular exploration.

## Introduction

Human autoimmune diseases are a group of pathologies that cause clinical damage or destruction of body tissue due to an immune response to its own antigens [1, 2]. There are many types of autoimmune diseases, such as SSC (systemic sclerosis), JIA (juvenile idiopathic arthritis), BD (Behcet’s disease), RA (rheumatoid arthritis), MS (multiple sclerosis), GD (Graves’ disease), SLE (systemic lupus erythematosus), and TID (type 1 diabetes) [1, 2]. A few cytokine genes have been reported to be linked to the autoimmune disease [2–4].

Interleukin 1 (IL1), including interleukin 1 alpha (α), beta (β) and receptor antagonist (ra), is a family of cytokines implicated in regulation of the inflammatory response and the incidence of clinical immune disease [5, 6]. The human interleukin 1 alpha (*IL1A*) gene, located on chromosome 2q13 [7], contains some common single nucleotide polymorphisms (SNPs), including rs1800587 (NM_000575.4:c.-949C>T)and rs17561 (NM_000575.4:c.340G>T), which have been reported to be linked to several autoimmune diseases in some populations [8–11]. However, negative conclusions have also been obtained by some studies [12–15].

Several meta-analyses have reported an association between *IL1A* rs17561, rs1800587 polymorphisms and the presence of various autoimmune diseases, including systemic lupus erythematosus [16, 17], rheumatoid arthritis [18], multiple sclerosis [19] and Graves’ disease [20]. However, the genetic relationship between *IL1A* SNPs and the risk of other autoimmune diseases, including systemic sclerosis and type 1 diabetes, has not been reported. In the present study, we probed the genetic role of *IL1A* gene SNPs rs17561 and rs1800587 in the risk of autoimmune diseases using quantitative synthesis of overall meta-analysis followed by subgroup analyses.

## Methods

The meta-analysis was conducted per the PRISMA (preferred reporting items for systematic reviews and meta-analyses) guidelines [21]. S1 File illustrates the meta-analysis on genetic association studies checklist, and S2 File shows the PRISMA 2009 checklist.

### Database searching

We obtained potentially suitable articles by systematically searching three databases (up to April 2018): PubMed, WOS (Web of Science), and Embase (Excerpta Medica Database). The search terms were shown in S3 File.

### Article screening

The following screening items were used to exclude publications: duplicates, reviews, letters, meta-analysis, abstracts or posters, and studies with unrelated data. Each study should have investigated an association between *IL1A* gene polymorphisms and autoimmune disease risk. The genotype frequency data could be extracted from both case and control groups. We also performed a chi-square-based Q-test to confirm that the genotype distribution of control group was consistent with HWE (Hardy-Weinberg Equilibrium).

### Data extraction

Detailed data, including the first author name, publication year, SNP, disease type, genotype frequency, genotyping assay, and ethnicity, were extracted and summarized independently. Conflicting data were discussed with all authors, and missing data were requested by e-mail. We also used the Newcastle-Ottawa Scale (NOS) system to assess the study quality and generate an NOS score. An NOS score < 5 means the study was poor quality, and such studies were excluded.

### Statistical association analysis

Stata/SE 12.0 software (StataCorp, USA) was used. To evaluate the strength of genetic relationships, *P*_*association*_, pooled ORs (odd ratios), and 95% CI (confidence interval) were generated referring to relevant publications [22–26]. The *P*_*association*_ value was then adjusted by the Bonferroni and false discovery rate (FDR) correction method [27], using R software version 3.4.3. Bonferroni and FDR-corrected *P*_association_ <0.05 from the association test was considered statistically significant. Six comparison models were utilized: allele T vs. G for rs17561, allele T vs. C for rs1800587 (allele); carrier T vs. G for rs17561, carrier T vs. C for rs1800587 (carrier); TT vs. GG for rs17561, TT vs. CC for rs1800587 (homozygote); GT vs. GG for rs17561, CT vs. CC for rs1800587 (heterozygote); GT+TT vs. GG for rs17561, CT+TT vs.CC for rs1800587 (dominant); TT vs. GG+GT for rs17561, and TT vs. CC+CT for rs1800587 (recessive). We also performed the subgroup analyses according to the characteristics of ethnicity, disease type, and control source.

Q statistics with *P*_heterogeneity_ (P value of heterogeneity) and I^2^ tests with I^2^ values were conducted to assess heterogeneity among the studies. When *P*_heterogeneity_ was >0.05 and the I^2^ value was <50%, the absence of high heterogeneity was inferred, and a fixed-effects model (Mantel-Haenszel method) was applied. Otherwise, a random-effects model (DerSimonian and Laird method) was utilized.

### Sensitivity analysis and bias evaluation

We performed sensitivity analysis to test whether the pooled results were stable. In sensitivity analysis, the effect of each study on the pooled ORs was evaluated as each included study was excluded one-by-one. We also performed Begg’s test and Egger’s test to evaluate publication bias. *P* values of Begg’s test and Egger’s test, namely *P*_Begg_ and *P*_Egger_, below 0.05 indicate the absence of publication bias.

## Results

### Study characteristics

**Fig 1:**
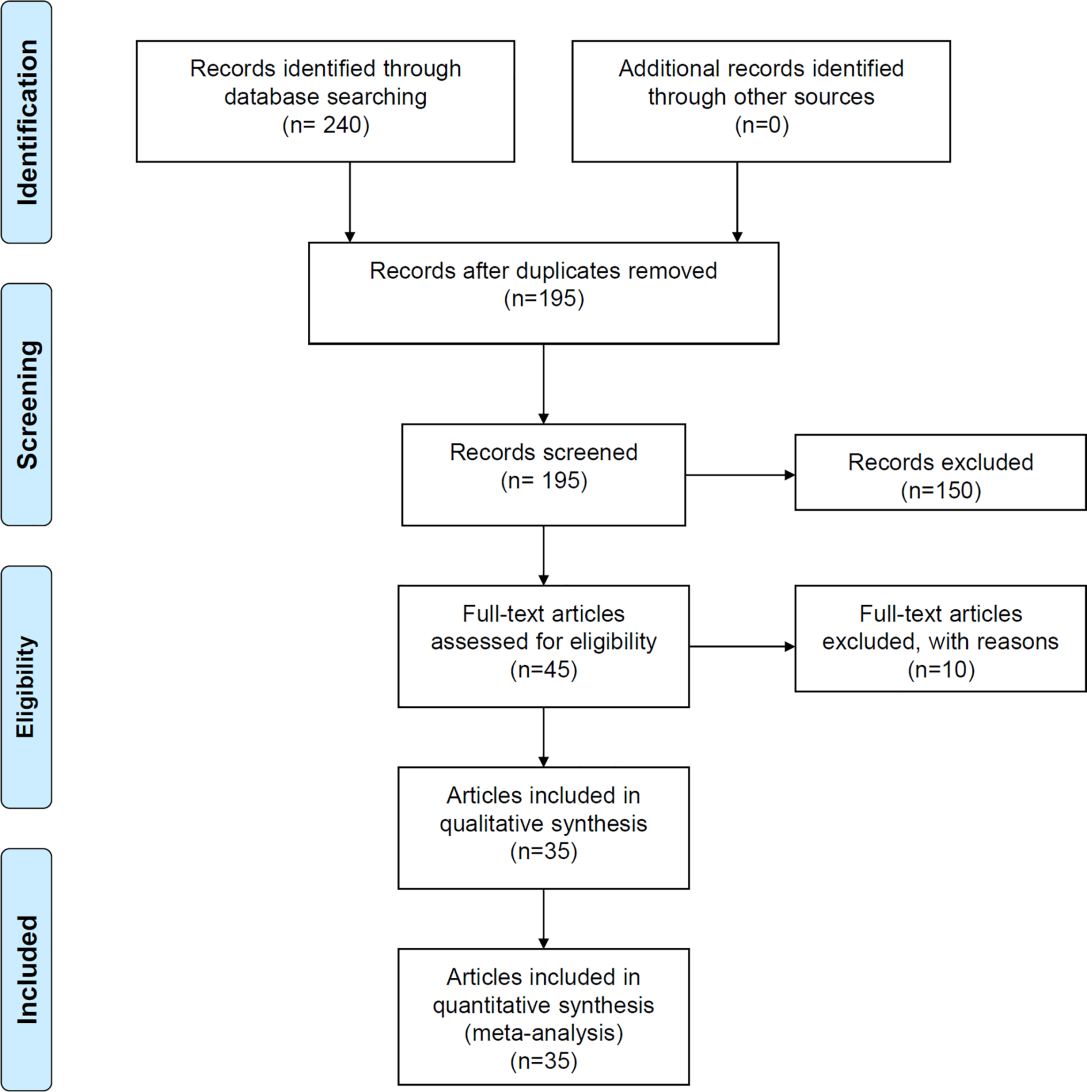
Flow diagram of database searching and article screening.

As shown in Fig 1, we searched three databases, identified a total of 240 articles [PubMed (n = 53), WOS (n = 81), Embase (n = 106)], and subsequently removed 45 duplicate articles. Then, 150 articles were excluded by our screening criteria. Assessing the eligibility of the remaining 45 articles, ten articles were removed, because seven did not contain complete genotype data and three were not consistent with HWE. Eventually, a total of 35 articles [8–15, 20, 28–53] were included, and none exhibited poor quality (all NOS score > 5). We list the characteristics of these studies in Table 1.

**Table 1:** Characteristics of eligible studies in meta-analysis.

First author, year	SNP	Disease	case			control			Source	Assay	NOS	Ethnicity
			AA	AB	BB	AA	AB	BB				
Abtahi, 2015	rs1800587	SSc	82	72	16	98	98	21	PB	PCR-SSP	seven	Asian
Aggarwal, 2012	rs1800587	JIA	42	47	5	93	78	14	PB	PCR-RFLP	seven	Asian
Akman, 2008	rs1800587	BD	32	17	4	19	22	7	PB	PCR-SSP Tray/Minitray and String Kits	seven	Caucasian
Beretta, 2007	rs1800587	SSc	117	70	17	112	76	16	PB	PCR-SSP	eight	Caucasian
Crilly, 2000	rs1800587	RA	45	47	7	33	22	5	PB	PCR-RFLP	six	Caucasian
Dominguez, 2017	rs1800587	RA	53	22	5	36	39	5	PB	PCR	eight	Caucasian
	rs17561	RA	55	21	4	47	29	4	PB	PCR	eight	Caucasian
Donn, 2001	rs1800587	JIA	183	125	22	105	113	18	PB	PCR-RFLP	six	Caucasian
Ferri, 2000	rs1800587	MS	189	177	33	198	203	38	PB	PCR-RFLP	eight	Caucasian
Genevay, 2002	rs17561	RA	105	101	24	76	60	8	PB	PCR	six	Caucasian
Harrison, 2008	rs1800587	RA	355	321	63	286	269	49	PB	PCR	eight	Caucasian
Havemose, 2007	rs1800587	JIA	5	3	2	14	10	1	PB	PCR-RFLP	seven	Caucasian
Havemose, 2007	rs1800587	RA	10	7	6	14	10	1	PB	PCR-RFLP	seven	Caucasian
Havemose, 2007	rs17561	JIA	5	3	2	14	10	1	PB	PCR-RFLP	seven	Caucasian
Havemose, 2007	rs17561	RA	10	7	6	14	10	1	PB	PCR-RFLP	seven	Caucasian
Hooper, 2003	rs1800587	MS	189	239	64	102	105	21	PB	PCR-RFLP	seven	Caucasian
Hutyrova, 2004	rs1800587	SSc	17	23	6	87	49	14	PB	PCR-SSP	seven	Caucasian
Johnsen, 2008	rs1800587	RA	687	507	89	546	445	105	PB	primer extension of multiplex products	eight	Caucasian
	rs17561	RA	686	513	87	545	443	104	PB	primer extension of multiplex products	eight	Caucasian
Kaijzel, 2002	rs17561	RA	194	171	31	117	79	22	PB	PCR-RFLP	seven	Caucasian
Kammoun, 2007	rs1800587	GD	89	42	0	188	37	0	PB	PCR-RFLP	six	African
Karasneh, 2003	rs1800587	BD	76	44	8	45	49	11	PB	gene sequencing	six	Caucasian
Kawaguchi, 2003	rs1800587	SSc	54	6	0	38	24	8	PB	gene sequencing	seven	Asian
	rs17561	SSc	54	6	0	30	30	10	PB	gene sequencing	seven	Asian
Khalilzadeh, 2009	rs1800587	GD	23	57	27	62	62	12	PB	PCR-SSP	seven	Asian
Kobayashi, 2007a	rs17561	RA	66	19	1	84	15	1	PB	PCR-RFLP	seven	Asian
Kobayashi, 2007b	rs17561	SLE	24	1	0	37	7	0	PB	PCR-RFLP	nine	Asian
Kobayashi, 2009	rs17561	RA	116	20	1	91	16	1	PB	PCR-RFLP	eight	Asian
Liu, 2010	rs1800587	GD	617	137	5	638	92	3	PB	GenomeLab SNPstream 12-plex Genotyping System	seven	Asian
Mann, 2002	rs1800587	MS	169	152	39	68	64	11	HB	PCR-RFLP	five	Caucasian
Mattuzzi, 2007	rs1800587	SSc	43	28	7	364	275	50	PB	Taqman MGB probes	seven	Caucasian
McDowell, 1995	rs1800587	RA	108	127	34	51	37	11	PB	gene sequencing	eight	Caucasian
Mirowska, 2011	rs17561	MS	106	107	15	87	90	16	PB	PCR-RFLP	six	Caucasian
Parks, 2004	rs1800587	SLE	62	57	25	18	43	12	PB	PCR-RFLP	seven	African
			43	32	11	68	109	25	PB	PCR-RFLP	seven	Caucasian
Pehlivan, 2011	rs1800587	ITP	53	18	0	67	4	0	PB	PCR-RFLP	eight	Caucasian
Sánchez, 2006	rs1800587	SLE	220	164	33	209	166	45	PB	gene sequencing	seven	Caucasian
Sarial, 2008	rs1800587	MS	33	66	1	62	62	12	PB	PCR-SSP	six	Asian
Tahmasebi, 2013	rs1800587	SLE	87	103	16	95	93	21	PB	PCR-SSP	seven	Asian
Zhou, 2016	rs1800587	TID	171	140	21	209	112	11	PB	TaqMan allelic discrimination assay	seven	Asian
		JIA	23	27	3	62	62	12	PB	PCR-SSP	six	Asian
Ziaee, 2014	rs1800587	SLE	26	25	7	62	62	12	PB	PCR-SSP	six	Asian

**Note:** SNP, single nucleotide polymorphisms; SSC, systemic sclerosis; JIA, juvenile idiopathic arthritis; BD, Behcet’s disease; RA, rheumatoid arthritis; MS, multiple sclerosis; GD, Graves’ disease; SLE, systemic lupus erythematosus; ITP, immune thrombocytopenic purpura; TID, type 1 diabetes; AA, major allele/major allele; AB, major allele/minor allele; BB, minor allele/minor allele; PB, population-based; HB, hospital-based; PCR-SSP, polymerase chain reaction with sequence-specific primers; PCR-RFLP, polymerase chain reaction-restriction fragment length polymorphism; NOS, Newcastle-Ottawa Scale.

### Meta-analysis of rs17561

**Fig 2:**
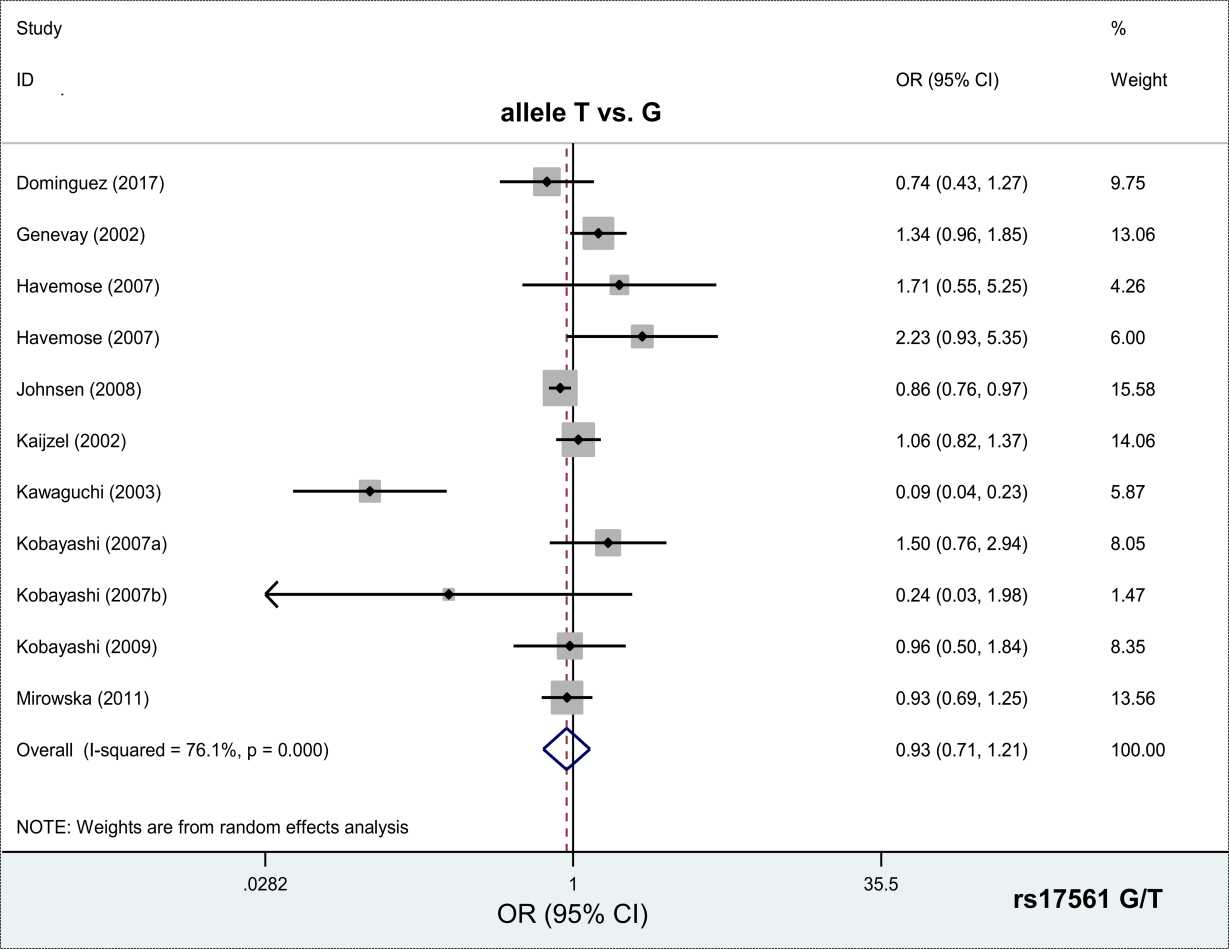
Meta-analysis of the *IL1A* rs17561 G/T polymorphism and the risk of autoimmune diseases under allele T vs. G model.

Eleven case-control studies with 2,561 cases and 2,099 controls were enrolled for the meta-analysis of the *IL1A* rs17561 G/T polymorphism. As shown in Table 2, compared with controls, no increased risk was detected in any of the cases under six comparison models, including allele T vs. G [*P*_*association*_ (*P* value in test of association) = 0.576, Bonferroni-corrected *P*_association_ = 1.000, FDR-corrected *P*_*association*_ = 0.703]; carrier T vs. G (*P*_*association*_ = 0.586, Bonferroni-corrected *P*_association_ = 1.000, FDR-corrected *P*_*association*_ = 0.703); TT vs. GG (*P*_*association*_ = 0.909, Bonferroni-corrected *P*_association_ = 1.000, FDR-corrected *P*_*association*_ = 0.909); GT vs. GG (*P*_*association*_ = 0.419, Bonferroni-corrected *P*_association_ = 1.000, FDR-corrected *P*_*association*_ = 0.703); GT+TT vs. GG (*P*_*association*_ = 0.438, Bonferroni-corrected *P*_association_ = 1.000, FDR-corrected *P*_*association*_ = 0.703); TT vs. GG+GT (*P*_*association*_ = 0.043, Bonferroni-corrected *P*_association_ = 1.000, FDR-corrected *P*_*association*_ = 0.258). Forest plot data of the meta-analyses under different models are provided in Fig 2 and S1–S5 Figs. We also performed subgroup analyses by ethnicity and disease types. Similar negative results were obtained under different comparison models (all *P*_*association*_>0.05, Bonferroni-corrected *P*_association_ >0.05, FDR-corrected *P*_*association*_>0.05, Table 3), except for the Asian (*P*_*association*_ = 0.024, Bonferroni-corrected *P*_association_ = 0.144, FDR-corrected *P*_*association*_ = 0.048) and PB (*P*_*association*_ = 0.043, Bonferroni-corrected *P*_association_ = 0.258, FDR-corrected *P*_*association*_ = 0.043) subgroups under the TT vs. GG+GT model. These data suggested that the *IL1A* rs17561 G/T polymorphism seems not be related to a risk for autoimmune disease overall.

**Table 2:** Meta-analysis of *IL1A* rs17561 G/T and rs1800587 C/T polymorphism.

SNP	Genetic models	N	Case/Control	*P*_association_	*P*_association_^&^	*P*_association_^#^	ORs (95% CIs)	I^2^ (%)	*P*_heterogeneity_	F/R	*P*_Begg_	*P*_Egger_
rs17561	allele T vs. G	11	2,561/2,099	0.576	1.000	0.703	0.93 (0.71, 1.21)	76.1	<0.001	R	1.000	0.950
	carrier T vs. G	11	2,561/2,099	0.586	1.000	0.703	0.94 (0.75, 1.18)	56.2	0.011	R	0.876	0.724
	TT vs. GGxs	10	2,536/2,055	0.909	1.000	0.909	0.97 (0.59, 1.59)	51.4	0.029	R	1.000	0.368
	GT vs. GG	11	2,561/2099	0.419	1.000	0.703	0.89 (0.67, 1.18)	64.4	0.002	R	0.161	0.393
	GT+TT vs. GG	11	2,561/2,099	0.438	1.000	0.703	0.88 (0.65, 1.21)	72.2%	<0.001	R	0.640	0.668
	TT vs. GG+GT	10	2,536/2,055	0.043	0.258	0.258	0.79 (0.64, 0.99)	44.9	0.060	F	0.858	0.289
rs1800587	allele T vs. C	31	7,381/4,049	0.548	1.000	0.860	1.04 (0.92, 1.18)	76.5	<0.001	R	0.634	0.396
	carrier T vs. C	31	7,381/4,049	0.546	1.000	0.860	1.03 (0.93, 1.14)	54.7	<0.001	R	0.683	0.502
	TT vs. CC	29	7.179/3,794	0.860	1.000	0.860	1.02 (0.81, 1.28)	54.7	<0.001	R	1.000	0.499
	CT vs. CC	31	7,381/4,049	0.747	1.000	0.860	1.03 (0.87, 1.21)	74.7	<0.001	R	0.919	0.915
	CT+TT vs. CC	31	7,381/4,049	0.672	1.000	0.860	1.04(0.88, 1.22)	77.4	<0.001	R	0.734	0.662
	TT vs. CC+CT	29	7.179/3794	0.698	1.000	0.860	1.04(0.86, 1.25)	38.2	0.020	R	0.866	0.481

**Note:** SNP, single nucleotide polymorphisms; N, number of case-control study; ORs, odd ratios; CIs, confidence intervals; *P*_*association*_, *P* value of association test; &, Bonferroni-corrected *P*_association_ value; #, FDR-corrected *P*_association_ value; F, fixed; R, random.

**Table 3:** Subgroup analysis of *IL1A* rs17561 G/T polymorphism.

Genetic models	subgroup	N	Case/Control	*P*_association_	*P*_association_^&^	*P*_association_^#^	ORs (95% CIs)	I^2^(%)	*P*_heterogeneity_
Allele T vs. G	Caucasian	7	2,253/1,777	0.811	1.000	0.811	1.02 (0.85, 1.24)	53.4	0.045
	Asian	4	308/322	0.258	1.000	0.516	0.94 (0.75, 1.18)	88.9	<0.001
	RA	7	2,238/1,767	0.593	1.000	0.625	1.06 (0.86, 1.32)	55.9	0.035
	PB	11	2,561/2,099	0.576	1.000	0.576	0.93 (0.71, 1.21)	76.1	<0.001
carrier T vs. G	Caucasian	7	2,253/1,777	0.493	1.000	0.493	0.96 (0.86, 1.07)	0.0	0.570
	Asian	4	308/322	0.279	1.000	0.493	0.54 (0.18, 1.64)	81.9	0.001
	RA	7	2,238/1,767	0.612	1.000	0.790	0.97 (0.87, 1.09)	0.0	0.448
	PB	11	2,561/2,099	0.586	1.000	0.586	0.94 (0.75, 1.18)	56.2	0.011
TT vs. GG	Caucasian	7	2,253/1,777	0.851	1.000	0.851	1.05 (0.65, 1.69)	54.0	0.043
	Asian	3	283.278	0.355	1.000	0.710	0.30 (0.02, 3.79)	58.4	0.090
	RA	7	2,238/1,767	0.882	1.000	0.882	1.04 (0.63, 1.72)	45.5	0.088
	PB	10	2,536/2,055	0.909	1.000	0.909	0.97 (0.59, 1.59)	51.4	0.029
GT vs. GG	Caucasian	7	2,253/1,777	0.805	1.000	0.805	0.98 (0.86, 1.12)	0.0	0.444
	Asian	4	308/322	0.280	1.000	0.560	0.50 (0.14, 1.77)	85.3	0.000
	RA	7	2,238/1,767	0.694	1.000	0.904	1.04 (0.86, 1.25)	18.7	0.287
	PB	11	2,561/2,099	0.419	1.000	0.419	0.89 (0.67, 1.18)	64.4	0.002
GT+TT vs. GG	Caucasian	7	2,253/1,777	0.984	1.000	0.984	1.00 (0.84, 1.19)	24.9	0.239
	Asian	4	308/322	0.262	1.000	0.524	0.45 (0.11, 1.82)	88.3	0.000
	RA	7	2,238/1,767	0.657	1.000	0.772	0.45 (0.11, 1.82)	37.8	0.141
	PB	11	2,561/2,099	0.438	1.000	0.438	0.88 (0.65, 1.21)	72.2	0.000
TT vs. GG+GT	Caucasian	7	2,253/1,777	0.125	0.750	0.125	0.84 (0.67, 1.05)	52.2	0.051
	Asian	3	283.278	0.024	0.144	0.048	0.21 (0.05, 0.81)	40.9	0.184
	RA	7	2,238/1,767	0.123	0.738	0.220	0.83 (0.65, 1.05)	41.2	0.116
	PB	10	2,536/2,055	0.043	0.258	0.043	0.79 (0.64, 0.99)	44.9	0.060

**Note:** RA, rheumatoid arthritis; PB, population-based control; ORs, odd ratios; CIs, confidence intervals; *P*_*association*_, *P* value of association test; &, Bonferroni-corrected *P*_association_ value; #, FDR-corrected *P*_association_ value; *P*_heterogeneity_, *P* value of heterogeneity.

### Meta-analysis of rs1800587

**Fig 3:**
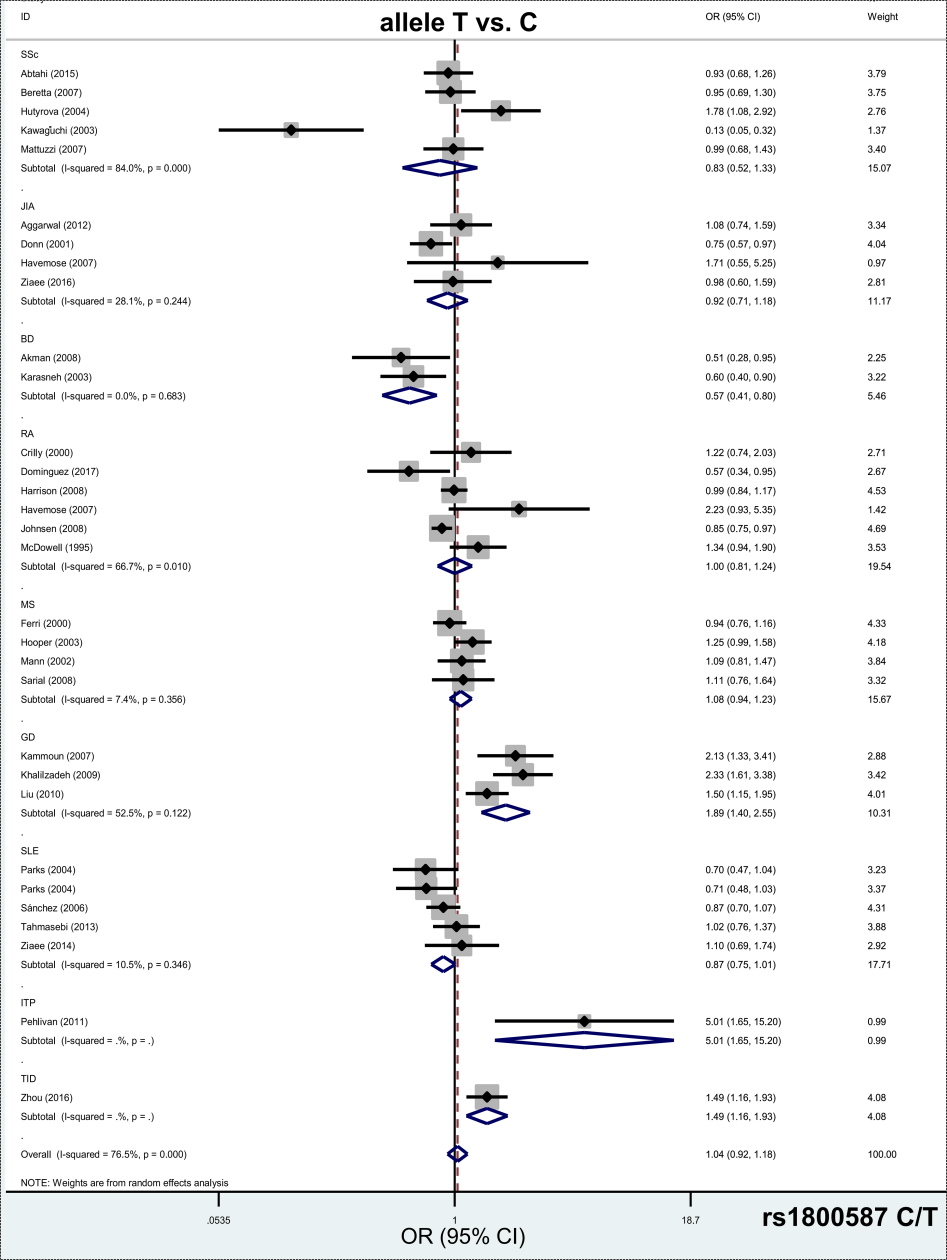
Subgroup analysis by disease type of the association between *IL1A* rs1800587 C/T polymorphism and the risk of autoimmune diseases under allele T vs. C model.

A total of 31 case-control studies with 7,381 cases and 4,049 controls were used for meta-analysis of the *IL1A* rs1800587 C/T Polymorphisms. Pooled data from the overall population (Table 2) presented the negative results under all comparison models (all *P*_*association*_ >0.05, Bonferroni-corrected *P*_association_ >0.05, FDR-corrected *P*_*association*_>0.05). Nevertheless, the data from the GD (Graves’ disease) subgroup analysis (Table 4), comprising three studies, showed an increased risk in cases of autoimmune diseases compared with controls under the genetic models of allele T vs. C (*P*_*association*_<0.001, Bonferroni-corrected *P*_association_ <0.006, FDR-corrected *P*_*association*_ <0.006, OR = 1.89, 95% CIs = 1.40, 2.55), carrier T vs. C (*P*_*association*_<0.001, Bonferroni-corrected *P*_association_ <0.006, FDR-corrected *P*_*association*_ <0.006, OR = 1.60, 95% CIs = 1.30, 1.98), CT vs. CC (*P*_*association*_<0.001, Bonferroni-corrected *P*_association_ <0.006, FDR-corrected *P*_*association*_ <0.006, OR = 1.94, 95% CIs = 1.38, 2.72), CT+TT vs.CC (*P*_*association*_ = 0.001, Bonferroni-corrected *P*_association_ = 0.006, FDR-corrected *P*_*association*_ = 0.006, OR = 2.12, 95% CIs = 1.38, 3.25). We did not observe a positive association between case and control groups in other subgroup analyses (Table 4, all *P*_*association*_>0.05). Fig 3 and S6–S8 Figs show the forest plots of the subgroup analysis by disease type under the models of allele T vs. C, carrier T vs. C, CT+TT vs.CC and CT vs. CC, respectively. S9 and S10 Figs show the forest plot data of subgroup analysis by ethnicity and control source under allele models. Based on the above data, the C/T genotype of *IL1A* rs1800587 C/T polymorphism is more likely to be statistically associated with an increased risk of Graves’ disease, but not other autoimmune diseases, such as systemic sclerosis, juvenile idiopathic arthritis, rheumatoid arthritis, multiple sclerosis and systemic lupus erythematosus.

**Table 4:** Subgroup analysis of *IL1A* rs1800587 C/T polymorphism.

Genetic models	subgroup	N	Case/Control	*P*_association_	*P*_association_^&^	*P*_association_^#^	ORs (95% CIs)	I^2^(%)	*P*_heterogeneity_
allele T vs. C	Asian	10	1,939/1,419	0.465	1.000	0.733	1.10 (0.85, 1.43)	80.5	<0.001
	Caucasian	19	5,167/2,424	0.612	1.000	0.733	0.97 (0.85, 1.10)	65.5	<0.001
	SSc	5	558/699	0.441	1.000	0.593	0.83 (0.52, 1.33)	84.0	<0.001
	JIA	4	487/274	0.494	1.000	0.593	0.92 (0.71, 1.18)	28.1	0.244
	RA	6	2,493/966	0.980	1.000	0.980	1.00 (0.81, 1.24)	66.7	0.010
	MS	4	1,351/430	0.284	1.000	0.568	1.11 (0.76, 1.64)	7.4	0.356
	GD	3	997/888	<0.001	<0.006	<0.006	1.89 (1.40, 2.55)	52.5	0.122
	SLE	5	911/452	0.071	0.426	0.213	0.87 (0.75, 1.01)	10.5	0.346
	PB	30	7,021/3,981	0.582	1.000	0.582	1.04 (0.91, 1.18)	77.2	<0.001
carrier T vs. C	Asian	10	1,939/1,419	0.316	1.000	0.677	1.11 (0.90, 1.37)	61.8	0.005
	Caucasian	19	5,167/2,424	0.451	1.000	0.677	0.96 (0.87, 1.06)	30.6	0.101
	SSc	5	558/699	0.512	1.000	0.614	0.88 (0.59, 1.30)	71.5	0.007
	JIA	4	487/274	0.385	1.000	0.614	0.91 (0.73, 1.13)	0.0	0.576
	RA	6	2,493/966	0.680	1.000	0.680	0.97 (0.84, 1.12)	24.3	0.252
	MS	4	1,351/430	0.494	1.000	0.614	1.05 (0.91, 1.22)	0.0	0.721
	GD	3	997/888	<0.001	<0.006	<0.006	1.60 (1.30, 1.98)	0.0	0.518
	SLE	5	911/452	0.236	1.000	0.614	0.91 (0.78, 1.06)	0.0	0.718
	PB	30	7,021/3,981	0.568	1.000	0.787	1.03 (0.93, 1.15)	56.2	<0.001
TT vs. CC	Asian	10	1,939/1,419	0.746	1.000	0.746	1.11 (0.60, 2.04)	69.5	0.001
	Caucasian	18	5,096/2,357	0.651	1.000	0.746	0.95 (0.77, 1.18)	36.5	0.061
	SSc	5	558/699	0.893	1.000	0.893	1.04 (0.58, 1.86)	44.6	0.125
	JIA	4	487/274	0.334	1.000	0.668	0.78 (0.46, 1.30)	0.0	0.505
	RA	6	2,493/966	0.876	1.000	0.893	0.97 (0.66, 1.43)	46.0	0.099
	MS	4	1,351/430	0.660	1.000	0.893	1.13 (0.66, 1.91)	52.6	0.097
	GD	2	866/700	0.032	0.192	0.192	3.72 (1.12, 12.39)	54.8	0.137
	SLE	5	911/452	0.082	0.492	0.246	0.75 (0.55, 1.04)	0.0	0.773
	PB	28	6,819/3,726	0.963	1.000	0.963	1.01 (0.80, 1.27)	36.5	0.061
CT vs. CC	Asian	10	1,939/1,419	0.120	0.720	0.360	1.24 (0.94, 1.64)	69.2	0.001
	Caucasian	19	5,167/2,424	0.327	1.000	0.491	0.92 (0.78, 1.09)	64.4	<0.001
	SSc	5	558/699	0.513	1.000	0.770	0.84 (0.50, 1.42)	77.5	0.001
	JIA	4	487/274	0.795	1.000	0.883	0.94 (0.60, 1.48)	54.3	0.087
	RA	6	2,493/966	0.883	1.000	0.883	0.98 (0.74, 1.29)	64.0	0.016
	MS	4	1,351/430	0.351	1.000	0.702	1.15 (0.86, 1.53)	57.9	0.068
	GD	3	997/888	<0.001	<0.006	<0.006	1.94 (1.38, 2.72)	42.1	0.178
	SLE	5	911/452	0.166	1.000	0.498	0.76 (0.51, 1.12)	70.9	0.008
	PB	30	7,021/3,981	0.733	1.000	0.826	1.03 (0.87, 1.22)	75.5	<0.001
CT+TT vs. CC	Asian	10	1,939/1,419	0.237	1.000	0.650	1.20 (0.89, 1.63)	77.0	<0.001
	Caucasian	19	5,167/2,424	0.433	1.000	0.650	0.94 (0.79, 1.11)	67.3	<0.001
	SSc	5	558/699	0.473	1.000	0.710	0.81 (0.46, 1.43)	82.6	<0.001
	JIA	4	487/274	0.730	1.000	0.876	0.93 (0.62, 1.40)	48.4	0.121
	RA	6	2,493/966	0.963	1.000	0.963	0.99 (0.75, 1.31)	66.8	0.010
	MS	4	1,351/430	0.294	1.000	0.588	1.14 (0.89, 1.45)	45.5	0.138
	GD	3	997/888	0.001	0.006	0.006	2.12 (1.38, 3.25)	63.7	0.063
	SLE	5	911/452	0.139	1.000	0.417	0.78 (0.55, 1.09)	63.5	0.027
	PB	30	7,021/3,981	0.680	1.000	0.902	1.04 (0.88, 1.23)	78.1	<0.001
TT vs. CC+CT	Asian	10	1,939/1,419	0.948	1.000	0.948	1.02 (0.60, 1.73)	62.9	0.004
	Caucasian	18	5,096/2,357	0.684	1.000	0.948	0.97 (0.82, 1.14)	13.0	0.299
	SSc	5	558/699	0.786	1.000	0.794	1.06 (0.69, 1.63)	14.0	0.325
	JIA	4	487/274	0.503	1.000	0.794	0.84 (0.51, 1.39)	0.0	0.444
	RA	6	2,493/966	0.723	1.000	0.794	0.94 (0.67, 1.31)	35.0	0.174
	MS	4	1,351/430	0.794	1.000	0.794	1.08 (0.62, 1.86)	58.6	0.065
	GD	2	866/700	0.001	0.006	0.006	2.97 (1.54, 5.72)	0.0	0.349
	SLE	5	911/452	0.356	1.000	0.794	0.87 (0.64, 1.17)	0.0	0.688
	PB	28	6,819/3,726	0.823	1.000	0.794	1.02 (0.85, 1.24)	38.8	0.020

**Note:** SSC, systemic sclerosis; JIA, juvenile idiopathic arthritis; RA, rheumatoid arthritis; MS, multiple sclerosis

GD, Graves’ disease; SLE, systemic lupus erythematosus; PB, population-based control; ORs, odd ratios; CIs, confidence intervals.

*P*_*association*_, *P* value of association test; &, Bonferroni-corrected *P*_association_ value; #, FDR-corrected *P*_association_ value; *P*_heterogeneity_, *P* value of heterogeneity.

### Heterogeneity, bias and sensitivity

**Fig 4:**
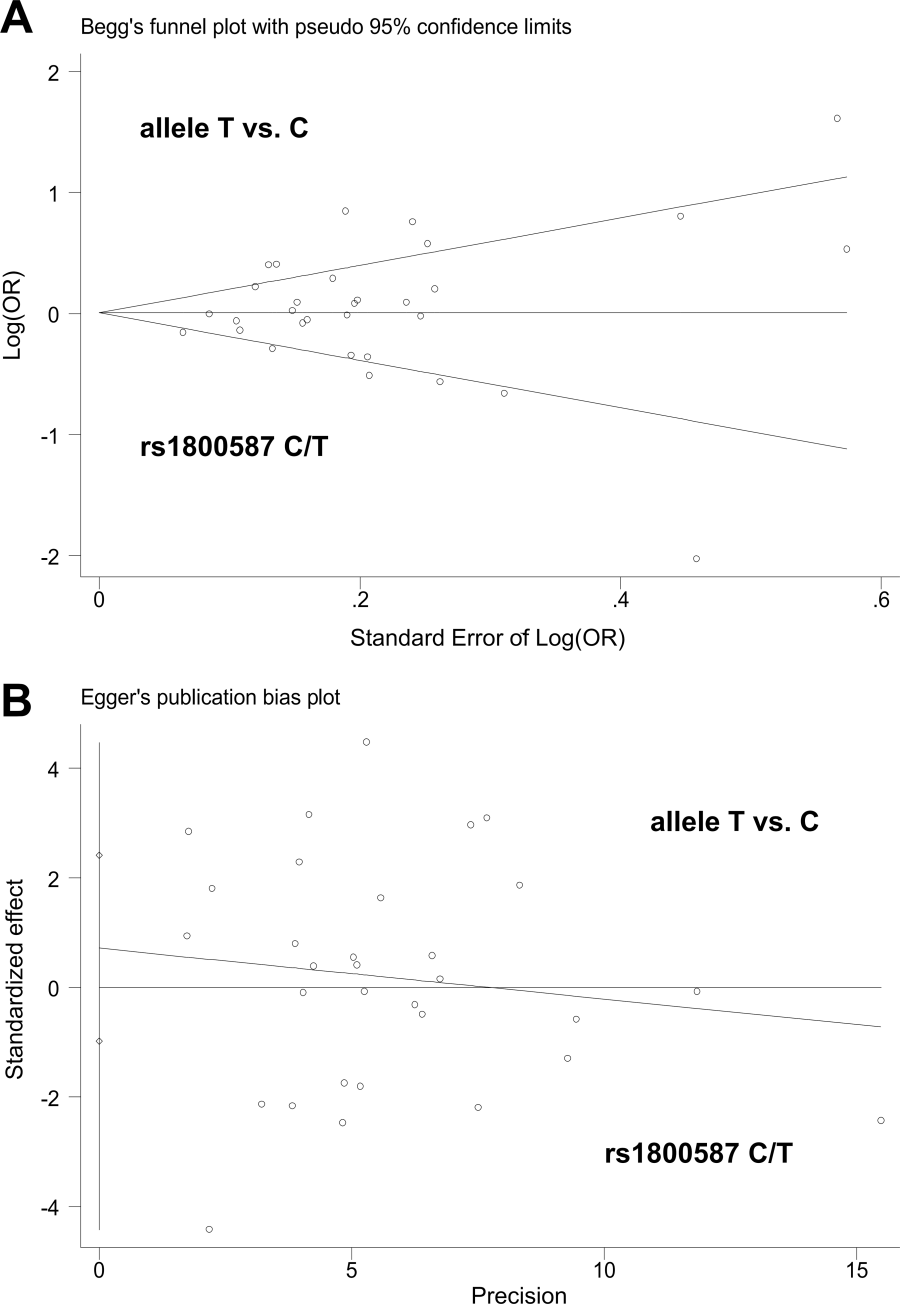
Begg’s test and Egger’s test for the allele T vs. C model of *IL1A* rs1800587 C/T polymorphism. (A) Begg’s test; (B) Egger’s test.

**Fig 5:**
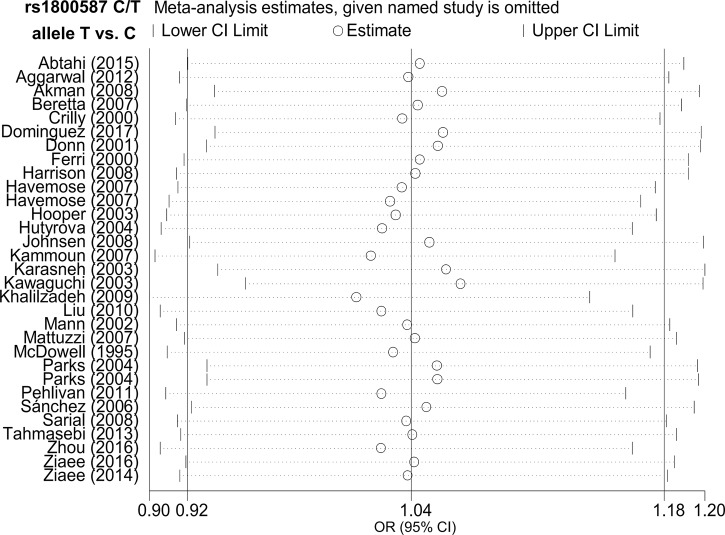
Sensitivity analysis for the allele T vs. C model of *IL1A* rs1800587 C/T polymorphism.

Apart from the TT vs. GG+GT comparison of rs17561, larger heterogeneity was detected (Table 2, I^2^ >50.0% or *P*_heterogeneity_ >0.05), and random effect models were utilized. In addition, as shown in Table 2, *P* value of Begg’s test and Egger’s test were >0.05 in all genetic models, indicating the absence of large publication bias. The plot data are shown in Fig 4 and S11 Fig. Furthermore, we believe our data are stable, because we did not observe any remarkable change of pooled ORs under any genetic models. The data for the allele T vs. C model of rs1800587 are shown in Fig 5, and other data are not shown.

## Discussion

Previously, the rs1800587 C/T SNP of *IL1A* gene was reported to not be linked to the risk or severity of systemic lupus erythematosus in a Spanish population [12], juvenile idiopathic arthritis in an Iranian population [15], and juvenile idiopathic arthritis in the UK [13]. *IL1A* rs17561 SNP was not associated with rheumatoid arthritis susceptibility in a Mexican population [14]. However, the *IL1A* rs1800587 and rs17561 SNPs were also reported to be associated with the risk of systemic sclerosis in a Japanese population [8]. The rs1800587 C/T SNP of *IL1A* gene has been related to susceptibility to systemic sclerosis in a Slovak Caucasian population [9], Graves’ ophthalmopathy in an Iranian population [10], and Graves’ disease in a Tunisian population [11]. Therefore, we first comprehensively explored the association between *IL1A* rs17561 and rs1800587 SNPs and the risk of overall autoimmune diseases using meta-analysis and subgroup analyses by characteristics of ethnicity, disease type and source of control.

In 2013, a meta-analysis was reported [17], investigating the genetic relationship between *IL1A* rs1800587 and rs17561 SNPs and the risk of systemic lupus erythematosus based on four case-control studies from three articles [12, 42, 48], which did not provide strong evidence for an association. In 2014, data from another meta-analysis containing four studies from three articles [12, 48, 51] supported a potential association for rs1800587 in Europeans [16]. In this study, we added another case-control study [53] to the subgroup meta-analysis of systemic lupus erythematosus for rs1800587, and observed a negative association.

In one meta-analysis of rheumatoid arthritis susceptibility[18], there were four case-control studies [32, 35, 38, 54] for rs1800587 and three case-control studies [34, 39, 43] for rs17561. No positive association between *IL1A* rs1800587 and rs17561 SNPs and the risk of rheumatoid arthritis was observed [18]. Here, we included more data for our updated meta-analysis and removed one case-control study [54], in which the genotype distribution of control group did not fulfill Hardy-Weinberg equilibrium. Seven case-control studies [14, 34, 36, 38, 39, 41, 43] were enrolled for the subgroup analysis of rs17561, and six case-control studies [14, 32, 35, 36, 38, 46] were used for rs1800587. Our pooled data with enhanced statistical power also indicated that the *IL1A* rs1800587 and rs17561 SNPs were not linked to the risk of rheumatoid arthritis, which was consistent with previous data [18].

Regarding multiple sclerosis susceptibility, in 2013, Huang et al. enrolled five case-control studies [33, 37, 44, 50, 55] for a meta-analysis of rs1800587 SNP and two case-control studies [47, 56] for meta-analysis of rs17561 SNP. However, negative association was reported for both s1800587 and rs17561 [19]. Here, due to the limitation of Hardy-Weinberg equilibrium, one case-control study [55] was excluded from our subgroup meta-analysis of rs1800587. We also found that the rs1800587 SNP was not linked to the risk of multiple sclerosis.

In 2010, Liu et al. investigated the genetic relationship between *IL1A* rs1800587 SNP and risk of Graves’ disease via a meta-analysis and found a positive association in an Asian population [20]. Here, our data in the subgroup meta-analysis of Graves’ disease showed similar results. It is possible that the rs1800587 SNP within the 5'-flanking regulatory region of *IL1A* gene affects the normal production, secretion or function of interleukin-1.

Some limitations exist in our meta-analysis. First, we did not obtain strong evidence regarding the effect of rs1800587 and rs17561 SNPs for the risk of different types of autoimmune diseases, due to the limited number of included independent case-control studies. Only two case-control studies [30, 40] were included in the subgroup of Graves’ disease under the homozygote and recessive models. Second, even though no remarkable publication bias was detected by our Begg’s test and Egger’s test, larger heterogeneity existed in the majority of comparisons. We observed a decreased level of heterogeneity in some subgroup analyses by disease type, such as the “rheumatoid arthritis, RA” subgroup of rs17561 and “multiple sclerosis, MS” subgroup of rs1800587. The factor of specific disease type may be involved in the source of heterogeneity. Further relevant researches with larger sample sizes were required. Third, we only acquired suitable case-control studies published in English. The outcome may be affected by the inclusion of unpublished articles, or articles published in another language. Fourth, it is worth analyzing the combined influence of different SNPs or cytokine genes, when more case-control studies become available.

Taken together, based on published articles in databases, our meta-analysis suggested that the rs1800587 polymorphism, rather than rs17561, within the *IL1A* gene, may be a genetic risk factor for Graves’ disease. However, *IL1A* rs17561 or rs1800587 polymorphism seems not to be statistically linked to the risk of other analyzed autoimmune diseases, such as systemic sclerosis, juvenile idiopathic arthritis, rheumatoid arthritis, multiple sclerosis and systemic lupus erythematosus.

## Supporting information

S1 Fig Meta-analysis of *IL1A* rs17561 G/T polymorphism and the risk of autoimmune diseases under carrier T vs. G model. (TIF)

S2 Fig Meta-analysis of *IL1A* rs17561 G/T polymorphism and the risk of autoimmune diseases under TT vs. GG model. (TIF)

S3 Fig Meta-analysis of *IL1A* rs17561 G/T polymorphism and the risk of autoimmune diseases under GT vs. GG model. (TIF)

S4 Fig Meta-analysis of *IL1A* rs17561 G/T polymorphism and the risk of autoimmune diseases under GT+TT vs. GG model. (TIF)

S5 Fig Meta-analysis of *IL1A* rs17561 G/T polymorphism and the risk of autoimmune diseases under TT vs. GG+GT model. (TIF)

S6 Fig Subgroup analysis by disease type of the association between *IL1A* rs1800587 C/T polymorphism and the risk of autoimmune diseases under carrier T vs. C model. (TIF)

S7 Fig Subgroup analysis by disease type of the association between *IL1A* rs1800587 C/T polymorphism and the risk of autoimmune diseases under CT+TT vs. CC model. (TIF)

S8 Fig Subgroup analysis by disease type of the association between *IL1A* rs1800587 C/T polymorphism and the risk of autoimmune diseases under CT vs. CC model. (TIF)

S9 Fig Subgroup analysis by ethnicity of the association between IL1A rs1800587 C/T polymorphism and the risk of autoimmune diseases under allele T vs. C model. (TIF)

S10 Fig Subgroup analysis by control source of the association between *IL1A* rs1800587 C/T polymorphism and the risk of autoimmune diseases under allele T vs. C model. (TIF)

S11 Fig Begg’s test and Egger’s test for the allele T vs. G model of *IL1A* rs17561 G/T polymorphism. (A) Begg’s test; (B) Egger’s test.
(TIF)

S1 File Meta-analysis of genetic association studies checklist. (DOCX)

S2 File PRISMA 2009 checklist. (DOC)

S3 File The search terms of database searching. (DOC)
