# Supplementary figures and images for: Meta-analyses of IL1A polymorphisms and the risk of several autoimmune diseases published in databases
Source: PLoS One. 2018 Jun 7;13(6):e0198693. doi: 10.1371/journal.pone.0198693 (PMC5991676; doi:10.1371/journal.pone.0198693)

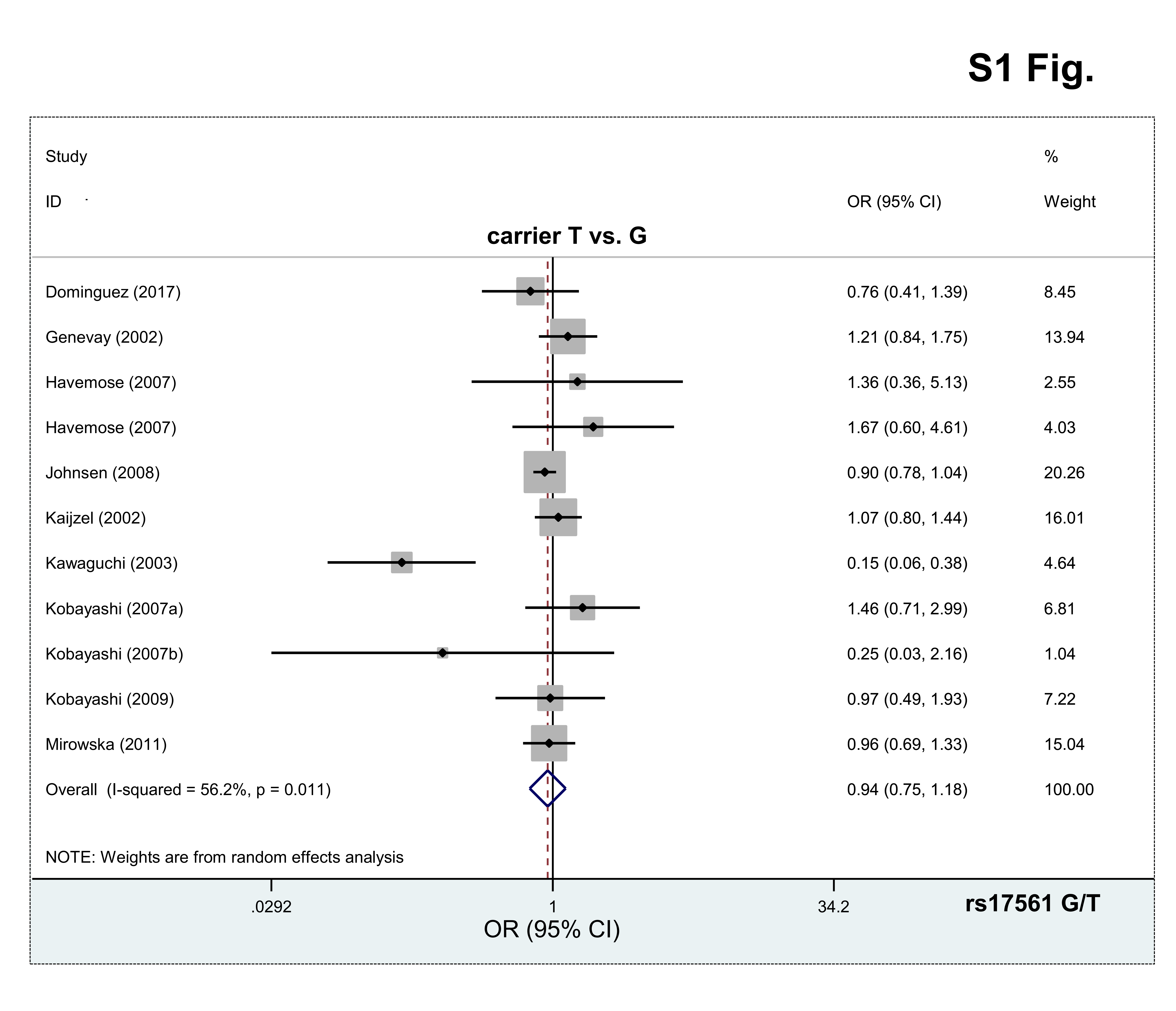

Supplement: S1 Fig — (TIF) [file pone.0198693.s001.tif]

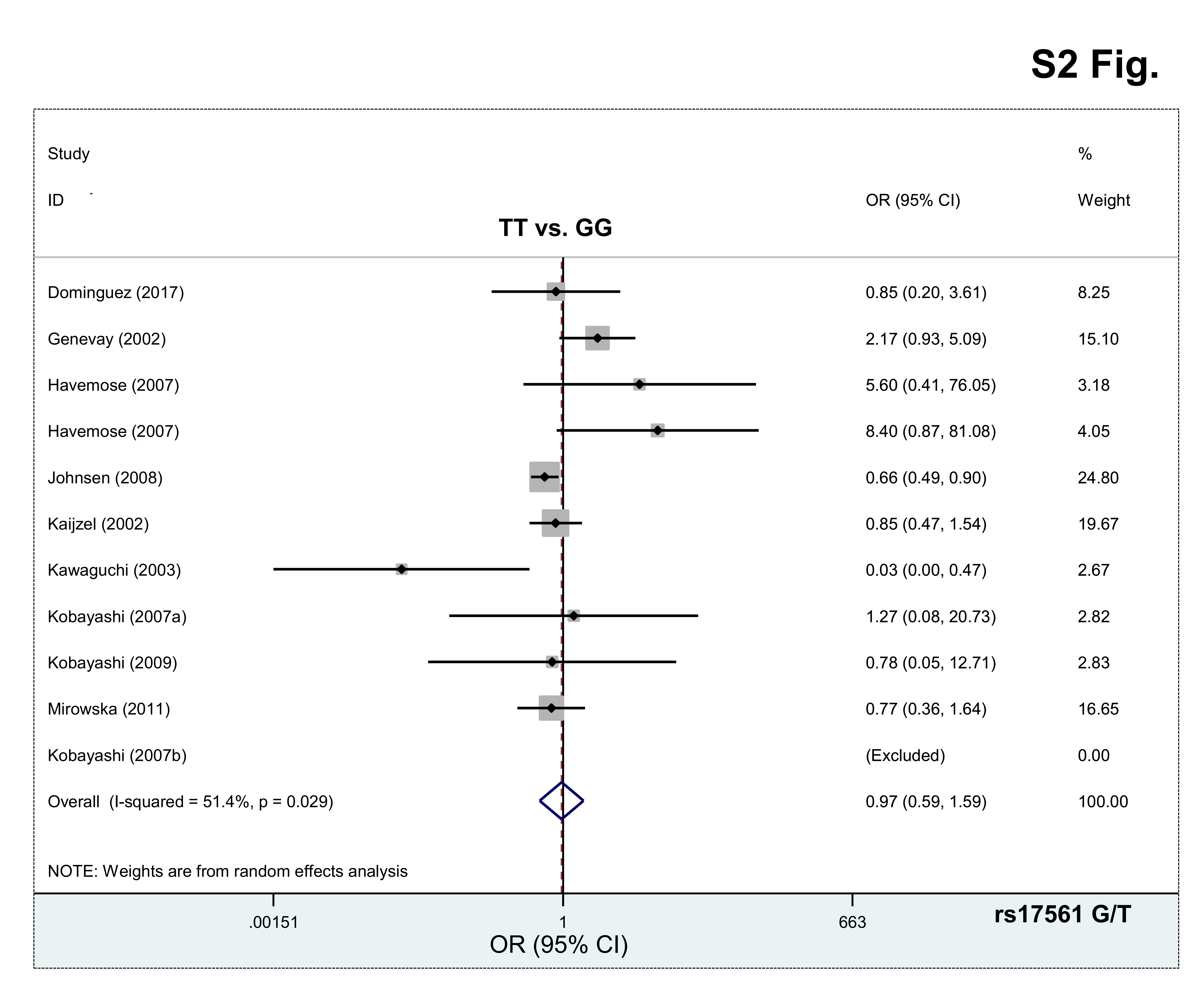

Supplement: S2 Fig — (TIF) [file pone.0198693.s002.tif]

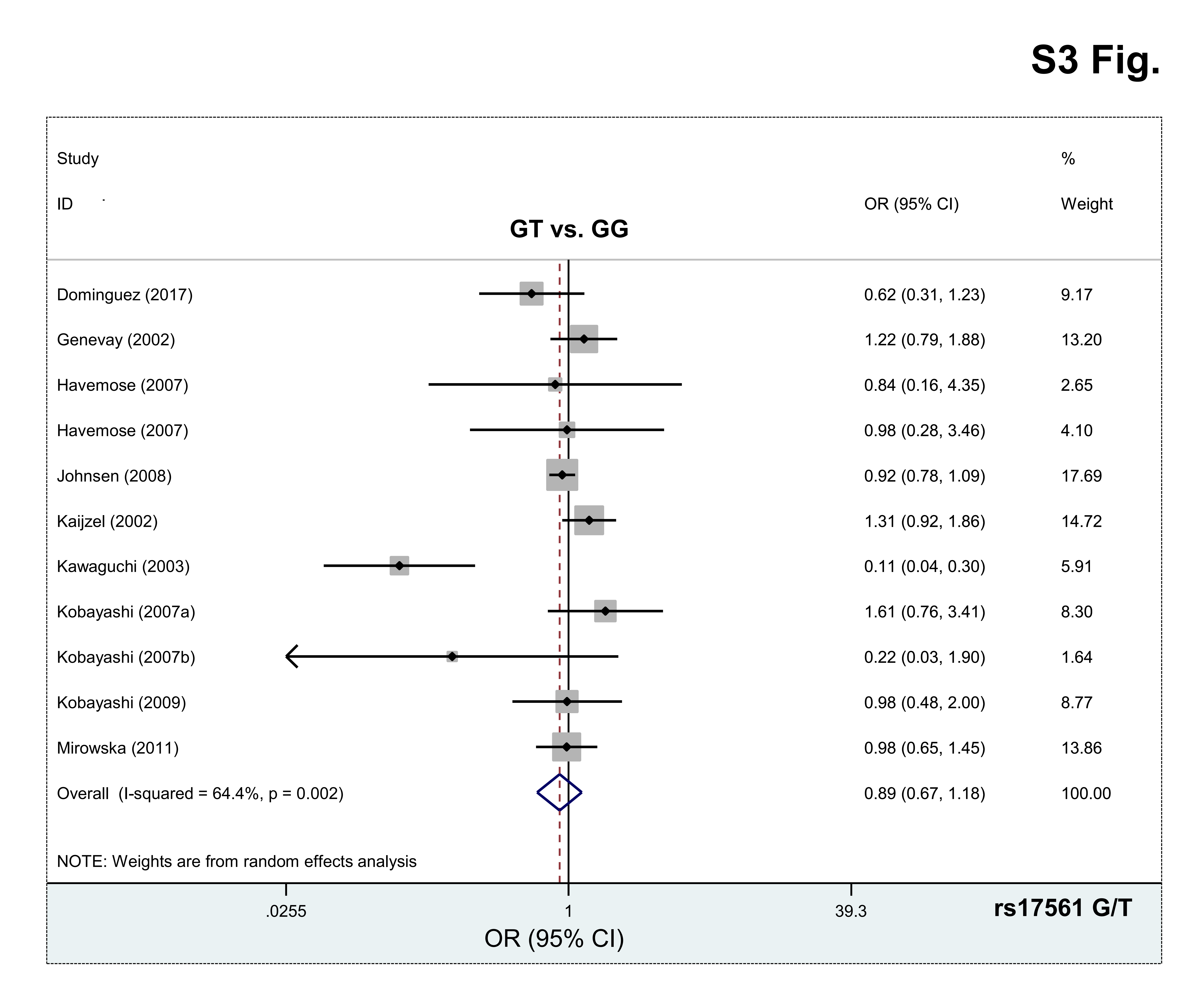

Supplement: S3 Fig — (TIF) [file pone.0198693.s003.tif]

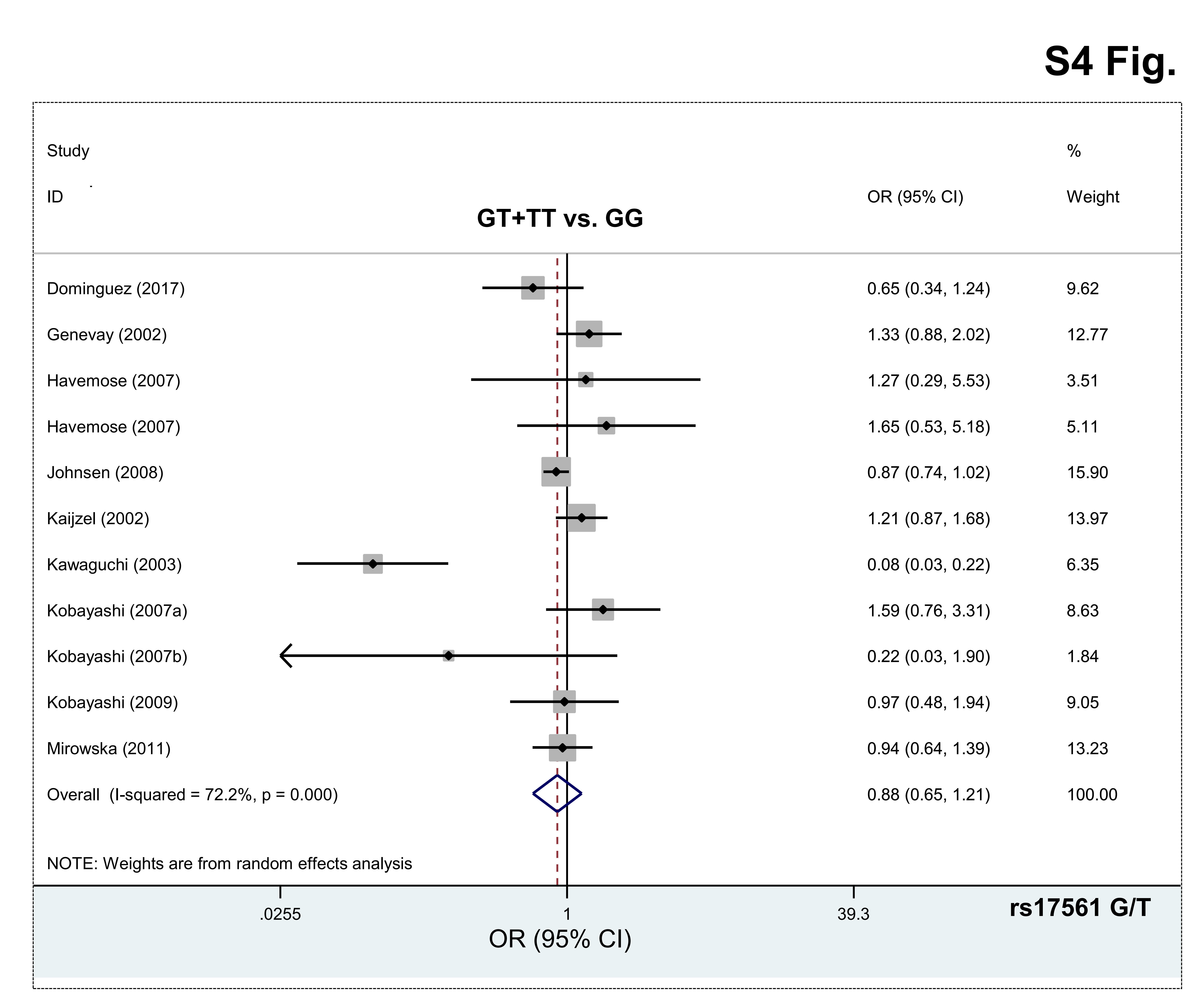

Supplement: S4 Fig — (TIF) [file pone.0198693.s004.tif]

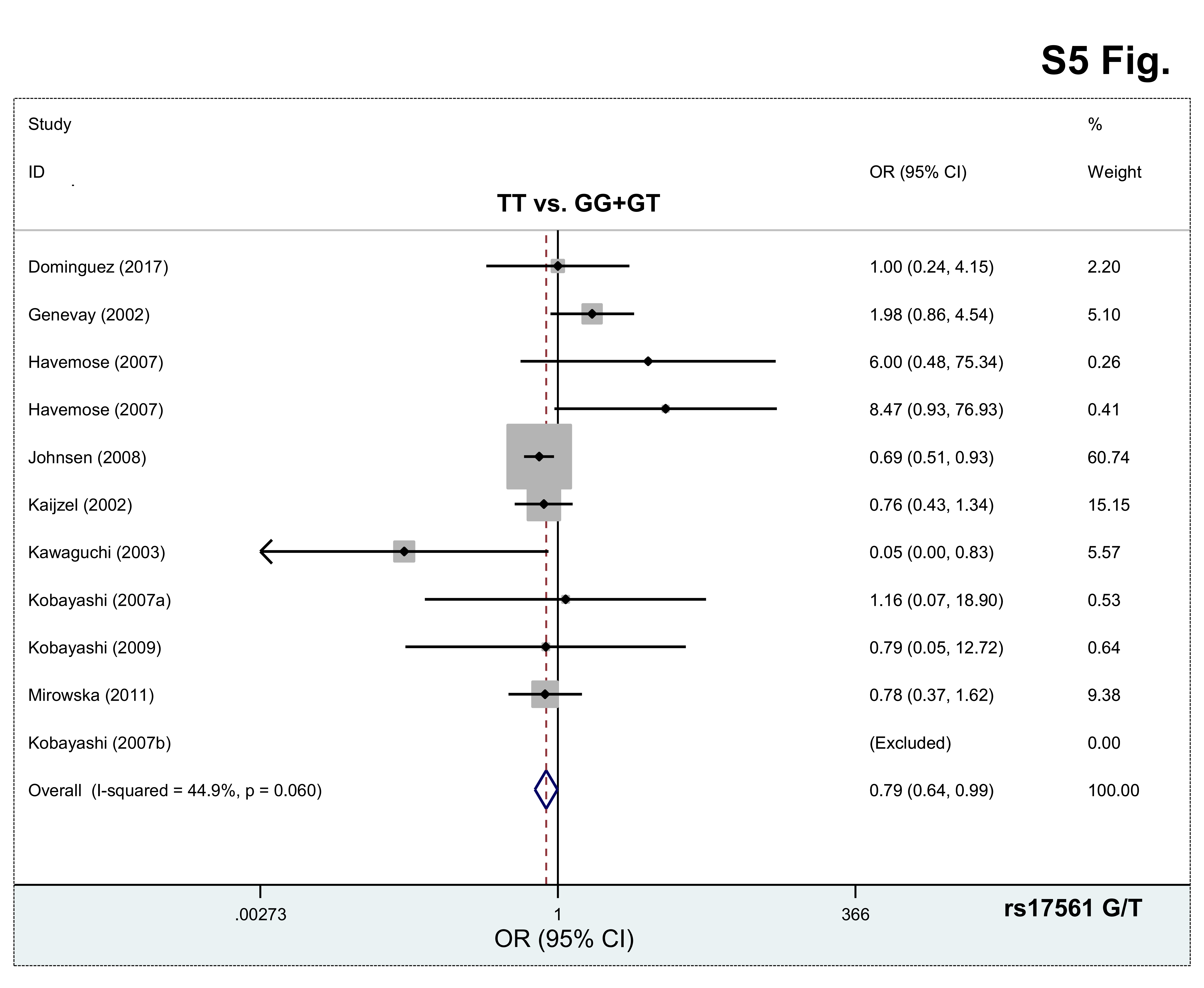

Supplement: S5 Fig — (TIF) [file pone.0198693.s005.tif]

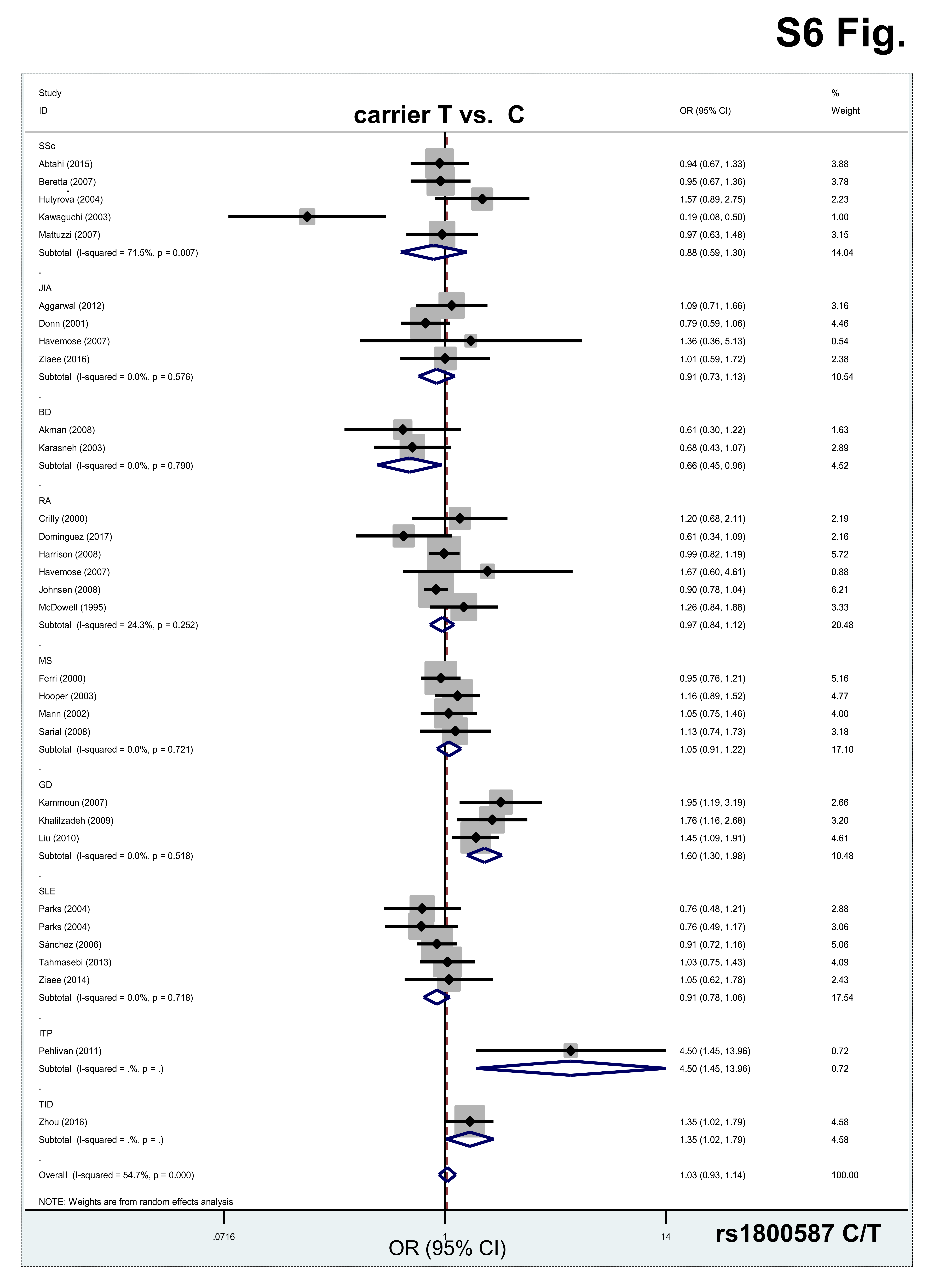

Supplement: S6 Fig — (TIF) [file pone.0198693.s006.tif]

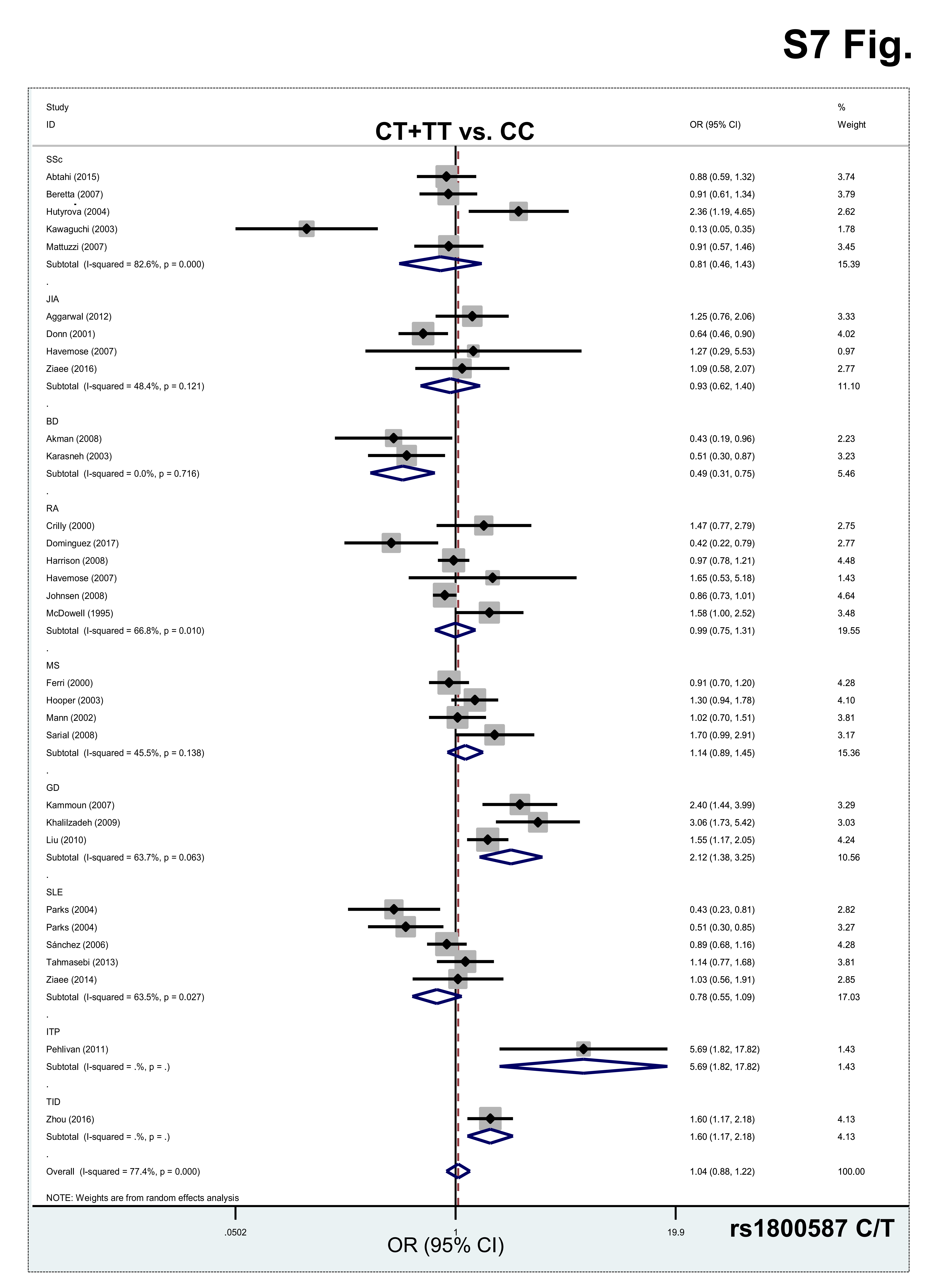

Supplement: S7 Fig — (TIF) [file pone.0198693.s007.tif]

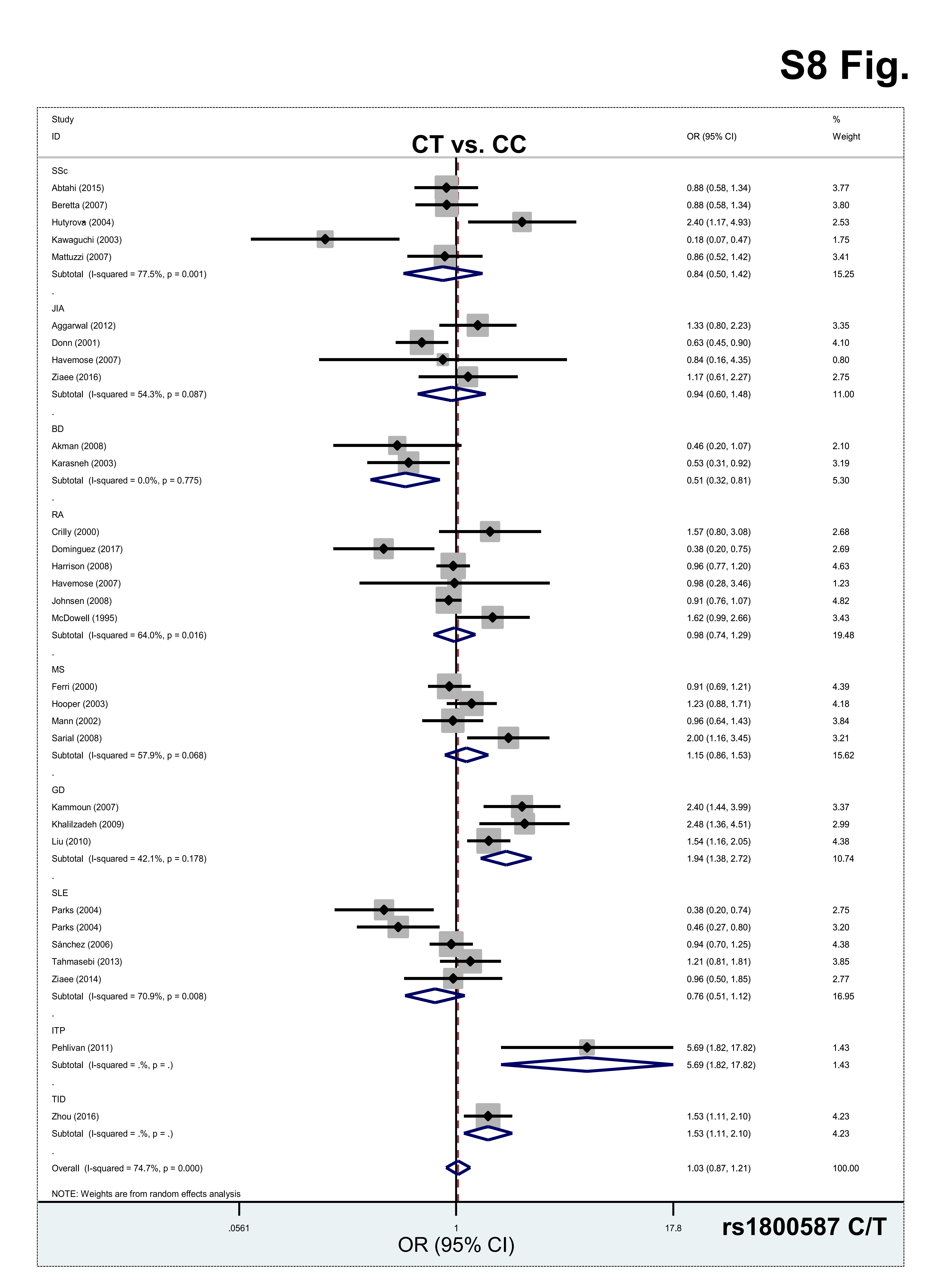

Supplement: S8 Fig — (TIF) [file pone.0198693.s008.tif]

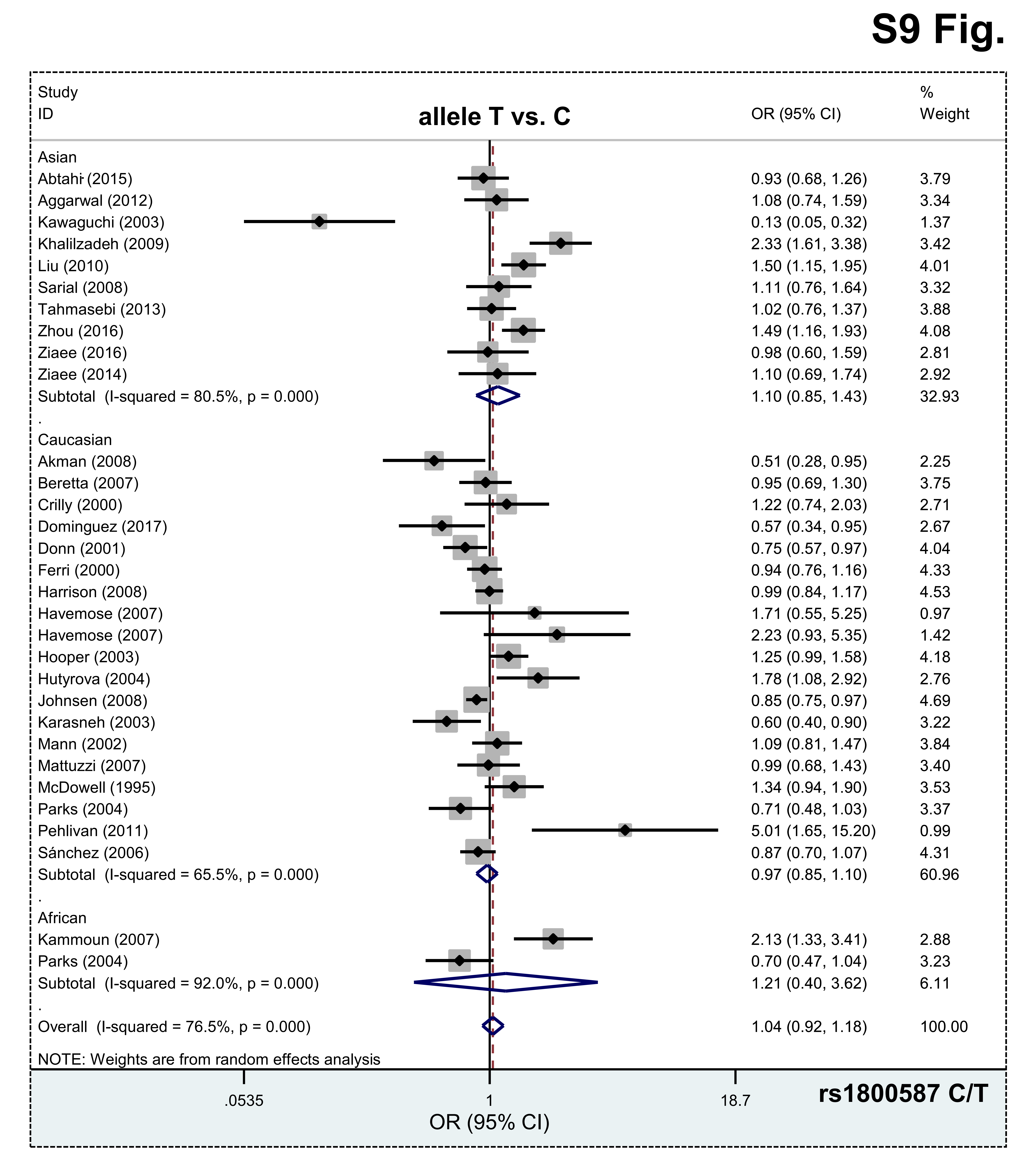

Supplement: S9 Fig — (TIF) [file pone.0198693.s009.tif]

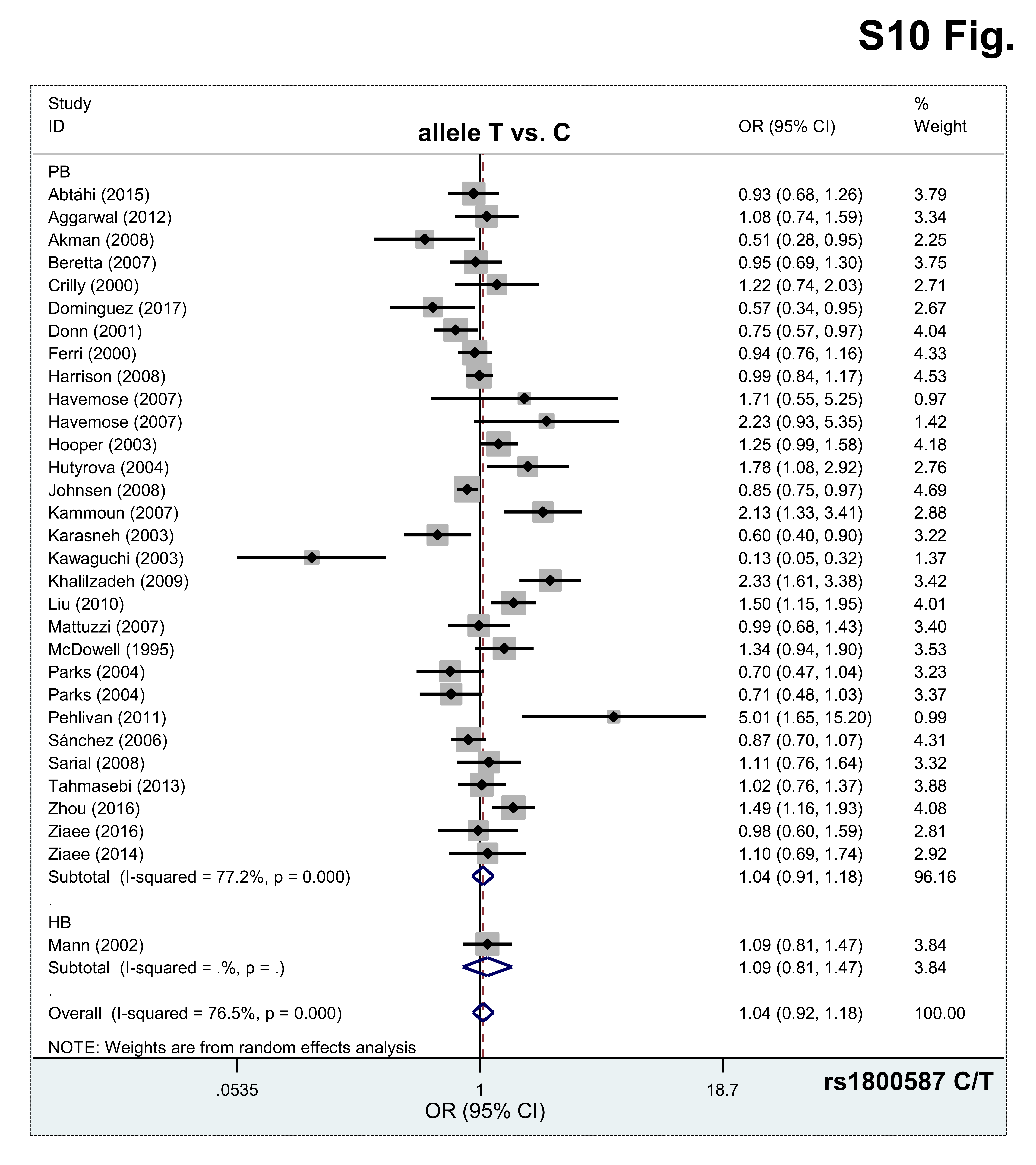

Supplement: S10 Fig — (TIF) [file pone.0198693.s010.tif]

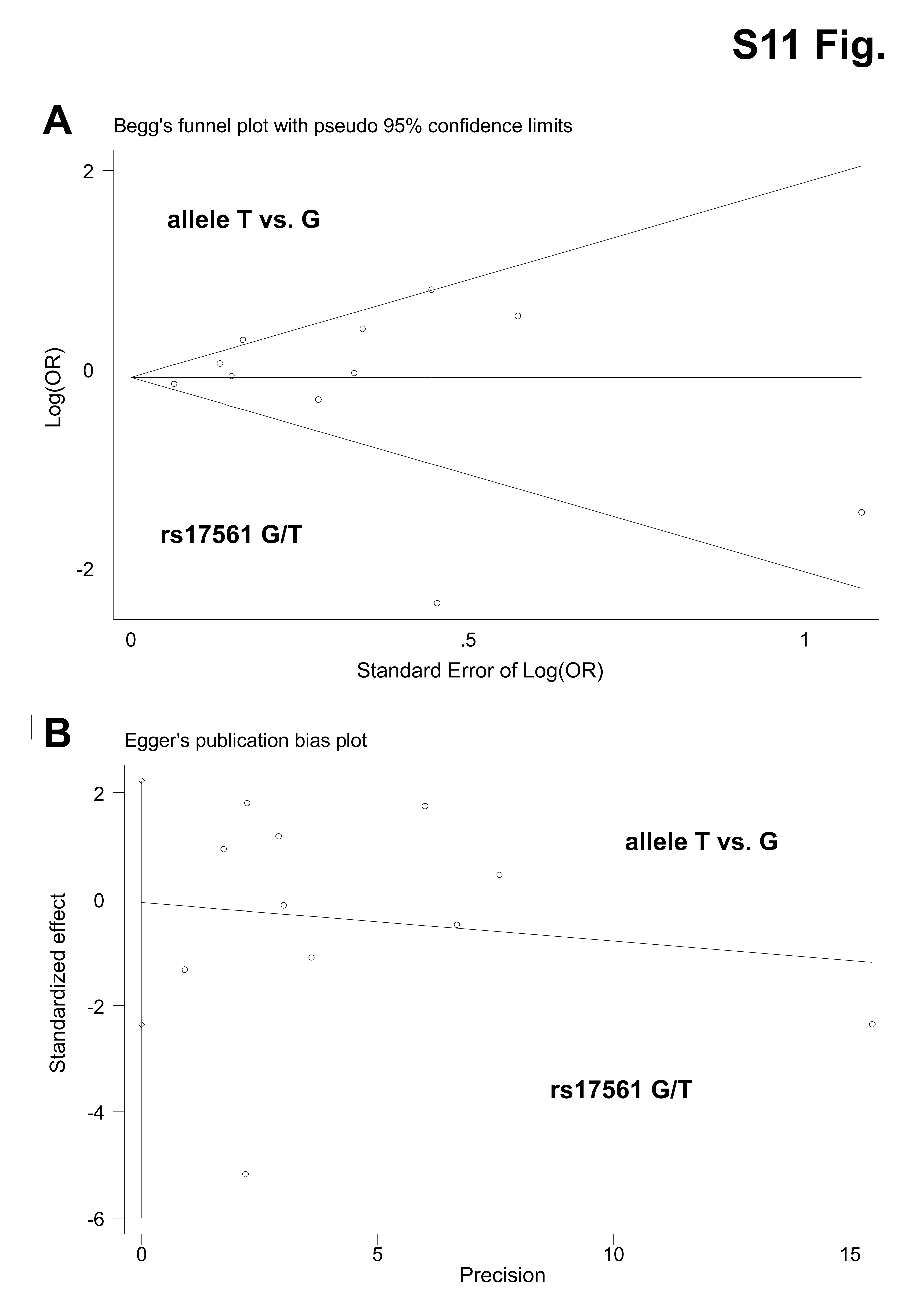

Supplement: S11 Fig — (A) Begg’s test; (B) Egger’s test. (TIF) [file pone.0198693.s011.tif]
