# Supplementary material for: Meta-analyses of IL1A polymorphisms and the risk of several autoimmune diseases published in databases
Source: PLoS One. 2018 Jun 7;13(6):e0198693. doi: 10.1371/journal.pone.0198693 (PMC5991676; doi:10.1371/journal.pone.0198693)
Supplement: S3 File — (DOC) [file pone.0198693.s014.doc]

| S3 Table The search terms of database searching. | | | |
| --- | --- | --- | --- |
| Database | **Set (#)** | Search terms | **Number of articles** |
| **PubMed** | | |  |
|  | #1 | (((((Autoimmune Diseases[MeSH Terms]) OR Disease, Autoimmune) OR Diseases, Autoimmune) OR Autoimmune Disease) OR Autoimmune Disorders) OR Autoimmune Disorder | 488,430 |
|  | #2 | (((((((((((((Interleukin-1alpha[MeSH Terms]) OR Interleukin 1alpha) OR IL-1 alpha) OR Interleukin-1 alpha) OR Interleukin 1 alpha) OR Hematopoietin-1) OR Hematopoietin 1) OR IL-1α) OR Interleukin-1α) OR ILA) OR IL1) OR IL-1A) OR IL1F1) OR IL1-ALPHA | 18,662 |
|  | #3 | ((((((Polymorphism, Single Nucleotide[MeSH Terms]) OR Nucleotide Polymorphism, Single) OR Nucleotide Polymorphisms, Single) OR Polymorphisms, Single Nucleotide) OR Single Nucleotide Polymorphisms) OR SNPs) OR Single Nucleotide Polymorphism | 125,666 |
|  | #4 | #1 AND #2 AND #3 | 53 |
| **WOS** | | |  |
|  | #1 | TS=(Autoimmune Diseases) OR TS=(Disease, Autoimmune) OR TS=(Diseases, Autoimmune) OR TS=(Autoimmune Disease) OR TS=(Autoimmune Disorders) OR TS=(Autoimmune Disorder) | 96,567 |
|  | #2 | TS=(Interleukin-1alpha) OR TS=(Interleukin 1alpha) OR TS=(IL-1 alpha) OR TS=(Interleukin-1 alpha) OR TS=(Interleukin 1 alpha) OR TS=(Hematopoietin-1) OR TS=(Hematopoietin 1) OR TS=(IL-1α) OR TS=(Interleukin-1α) OR TS=(IL1A) OR TS=(ILA) OR TS=(IL1) OR TS=(IL-1A) OR TS=(IL1F1) OR TS=(IL1-ALPHA) | 75,549 |
|  | #3 | TS=(Polymorphism, Single Nucleotide) OR TS=(Nucleotide Polymorphism, Single) OR TS=(Nucleotide Polymorphisms, Single) OR TS=(Polymorphisms, Single Nucleotide) OR TS=(Single Nucleotide Polymorphisms) OR TS=(SNPs) OR TS=(Single Nucleotide Polymorphism) OR TS=(SNP) | 113,934 |
|  | #4 | (#1 AND #2 AND #3) web of science core collection Timespan=1998-2018 | 81 |
| **Embase** | | |  |
|  | #1 | 'autoimmune disease'/exp/mj OR 'auto immune disease'/exp OR 'auto immune disease' OR 'auto immunologic disease'/exp OR 'auto immunologic disease' OR 'auto-immune disorder'/exp OR 'auto-immune disorder' OR 'auto-immune disorders'/exp OR 'auto-immune disorders' OR 'autoaggression, immune'/exp OR 'autoaggression, immune' OR 'autoaggressive disease'/exp OR 'autoaggressive disease' OR 'autoantibody disease'/exp OR 'autoantibody disease' OR 'autoimmune diseases'/exp OR 'autoimmune diseases' OR 'autoimmune disorder'/exp OR 'autoimmune disorder' OR 'autoimmune disorders'/exp OR 'autoimmune disorders' OR 'autoimmune disturbance'/exp OR 'autoimmune disturbance' OR 'autoimmune pathology'/exp OR 'autoimmune pathology' OR 'autoimmuno disease'/exp OR 'autoimmuno disease' OR 'autoimmunologic disease'/exp OR 'autoimmunologic disease' | 576,753 |
|  | #2 | 'interleukin 1alpha'/exp/mj OR 'hemopoietin 1'/exp OR 'hemopoietin 1' OR 'il 1 alpha'/exp OR 'il 1 alpha' OR 'il 1alpha'/exp OR 'il 1alpha' OR 'interleukin 1 alpha'/exp OR 'interleukin 1 alpha' OR 'interleukin-1alpha'/exp OR 'interleukin-1alpha' OR 'il1a' OR 'ila' OR 'il1' OR 'il-1a' OR 'il1f1' OR 'il1-alpha' | 22,934 |
|  | #3 | 'single nucleotide polymorphism'/exp/mj OR 'polymorphism, single nucleotide'/exp OR 'polymorphism, single nucleotide' OR 'single nucleotide variant'/exp OR 'single nucleotide variant' OR 'single nucleotide variation'/exp OR 'single nucleotide variation' | 149,444 |
|  | #4 | #1 AND #2 AND #3 | 106 |
